# Computer Vision-Based Characterization of Temporal Feeding Dynamic in Group-Housed Dairy Goats

**DOI:** 10.3390/ani16213390

**Published:** 2026-10-27

**Authors:** Yifeng Song, Qier Mu, Na Liu, Yuan Wang, Junqing Guo, Jingwei Qi, Xiaoping An

**Affiliations:** 1 College of Animal Science, Inner Mongolia Agricultural University, Hohhot 010018, China; 2 Key Laboratory of Smart Animal Husbandry at Universities of Inner Mongolia Autonomous Region, Research Center of Dairy Cattle Breeding and Farming Technology, National Center of Technology Innovation for Dairy Industry, Inner Mongolia Agricultural University, Hohhot 010018, China

**Keywords:** dairy goats, feeding behavior, computer vision, deep learning, feeding dynamics, digital behavioral phenotypes, precision livestock farming

## Abstract

Feeding behavior is closely related to nutrient intake, health, and production performance, yet its temporal dynamics are difficult to quantify in group-housed dairy goats. We developed a computer vision-based framework to characterize feeding dynamics under commercial farm conditions. Video data were collected from 550 Guanzhong dairy goats housed in five pens over three consecutive days. YOLO11, RF-DETR, and RT-DETR were compared for feeding-area goat detection, and temporal feeding features were analyzed to characterize high feeding activity (HFA) and low feeding activity (LFA). In a subsequent recording phase, head-down feeding behavior was assessed in 150 goats originating from the classified source pens. A total of 24 of these goats (12 HFA and 12 LFA) were selected for exploratory milk-production and serum comparisons. RF-DETR showed the best overall detection performance, with a precision (*P*) of 87.39%, recall (*R*) of 84.00%, *F*1-score of 85.66%, mean average precision (*mAP*)@0.5 of 90.38%, and *mAP*@0.5:0.95 of 53.30%. Goats exhibited a “rapid entry–peak aggregation–gradual departure” feeding pattern after feed delivery. Among the temporal features, rise time of feeding-area goat count (RTFGC), fall time of feeding-area goat count (FTFGC), and feeding-area goat count increase rate (FGCIR) were the most informative features for distinguishing HFA from LFA. HFA goats reached peak feeding-area occupancy earlier, entered the feeding area at a higher rate, and maintained feeding activity for longer than LFA goats. HFA goats also showed higher head-down feeding proportions, greater average daily dry matter intake (DMI), and higher milk yield, as well as distinct serum immune, antioxidant, and neuroendocrine-related profiles. These findings indicate that feeding dynamics derived from video observations can provide a quantitative digital behavioral phenotype for characterizing individual differences in dairy goat feeding activity and their associations with production and physiological status.

## Simple Summary

Feeding behavior provides important information about how much animals eat, their health, and their production, but it is difficult to measure continuously in large commercial herds. This study used video images and computer vision, which allows software to recognize animals in images, to describe changes in dairy goat presence in the feeding area after feed delivery. We compared three image-recognition methods and selected the best-performing method for counting goats. We summarized changes in feeding-area goat numbers over time to characterize higher- and lower-activity patterns at the pen level. A subsequent video assessment examined head-down feeding in selected goats from the original pens, followed by exploratory milk-production and serum comparisons in a smaller subset. Goats generally showed rapid entry, peak aggregation, and gradual departure after feeding. Goats with higher feeding activity entered sooner, entered faster, and remained active longer. They also had greater feed intake and milk yield and showed differences in selected immune, antioxidant, and hormone-related blood indicators. These findings suggest that feeding dynamics from video observations may provide a useful digital behavioral measure for evaluating individual differences and supporting precision livestock management.

## 1. Introduction

Feeding behavior is an important indicator of nutrient intake, health, and production performance in ruminants [1]. In dairy goats, feeding behavior is not simply characterized by feeding or non-feeding states but is influenced by feed availability, social competition, individual feeding motivation, and physiological status [1,2]. Consequently, feeding behavior exhibits substantial temporal dynamics and individual variation. Following feed delivery, dairy goats typically undergo a sequence of rapid entry into the feeding area, peak feeding activity, and gradual departure, with individuals differing in feeding initiation, persistence, and patterns of feeding-area occupancy [3]. Continuous and objective quantification of these behavioral dynamics may therefore provide valuable information for precision feeding, animal monitoring, and production management.

The development of precision livestock farming (PLF) has increased the demand for objective, continuous, and non-invasive monitoring of animal feeding behavior [4,5,6]. Conventional approaches, including direct observation, manual video analysis, and wearable sensors, are often labor-intensive and may be subject to observer bias [7,8,9], while contact-based devices may also interfere with normal animal behavior and increase equipment-maintenance requirements [4]. Computer vision and deep learning provide a non-contact alternative, enabling automated detection of animals and extraction of behavior-related information from video data [10,11]. Object detection algorithms, such as YOLO and DETR, can identify animals in complex farm environments and extract spatial and behavior-related information from video images [12,13,14,15,16]. Recent advances in artificial intelligence and PLF have further expanded the potential for automated monitoring of goat behavior, feeding activity, health, and production [17]. Recent studies have extended computer vision from basic animal detection toward more detailed behavioral monitoring. In small ruminants, lightweight deep-learning models have been developed to recognize feeding and other behavioral states while improving computational efficiency and potential deployment feasibility [14,18]. Video-based approaches have also begun to incorporate temporal information to characterize behavioral patterns in dairy goats [19]. In dairy cattle, computer vision has been combined with individual identification to quantify feeding time, duration, and frequency [15]. Collectively, these studies demonstrate substantial progress in behavior recognition, temporal modeling, and individual-level monitoring. However, recognizing predefined behavioral categories and quantifying interpretable feeding dynamics are related but distinct tasks. In particular, the extent to which continuous post-feeding occupancy trajectories can be transformed into meaningful temporal features, and how such pen-level patterns relate to subsequent individual feeding observations, remains less well characterized in group-housed dairy goats. Therefore, an important next step is to examine the relationship between video-derived feeding-area occupancy dynamics and individual feeding behavior, while further exploring their associations with production and physiological measures. This shifts the emphasis from detection performance alone toward the interpretation and potential practical relevance of video-derived behavioral measures.

This study aimed to evaluate the use of computer vision for quantifying feeding dynamics in group-housed dairy goats under commercial-farm conditions. Specifically, we sought to characterize temporal changes in feeding-area occupancy following feed delivery, assess their potential for distinguishing high feeding activity (HFA) from low feeding activity (LFA), and examine their correspondence with individual head-down feeding behavior. We also aimed to explore associations of these feeding-activity classifications with production performance and selected serum physiological indicators.

## 2. Materials and Methods

### 2.1. Animals, Experimental Site, and Feeding Management

This study was conducted at a large-scale commercial dairy goat farm located in Hohhot, Inner Mongolia Autonomous Region, China (40°48′ N, 111°39′ E). In total, 550 healthy Guanzhong dairy goats were enrolled in this study and housed in five standardized pens, each measuring 110 m in length, 11.5 m in width, and 6 m in height. The study followed a sequential sampling design. Group-level feeding dynamics were first evaluated in the five original pens. Following pen-level classification, 150 goats were selected for a subsequent individual behavioral observation phase, comprising 75 goats from HFA-classified pens and 75 from LFA-classified pens. These goats had body condition scores (BCS) of 2.75–3.25, parities of 2–4, and approximately 120 days in milk. A further subset of 24 goats, comprising 12 from each pen category, was selected for exploratory milk-production and serum assessments. This subset had BCS values of 2.75–3.00 and 120 ± 10 days in milk. All animals remained in their original pens, with no regrouping associated with the study classifications.

All goats were managed according to the routine management practices of the farm and received the same feeding and husbandry conditions, including a uniform basal diet and feeding schedule, regular health examinations and vaccination, and appropriate environmental management. Goats had ad libitum access to feed and fresh drinking water. Feed was offered twice daily at 09:00 and 14:00, with a target daily feed refusal rate of approximately 5% to ensure sufficient feed availability in the feeding area. The composition and nutrient levels of the basal diet are presented in Table 1. All experimental procedures were conducted in accordance with institutional guidelines for the care and use of experimental animals and were reviewed and approved by the Animal Welfare and Ethics Committee of Inner Mongolia Agricultural University on 19 December 2023 (Approval No. NND2023117).

### 2.2. Video Acquisition

**Figure 1:**
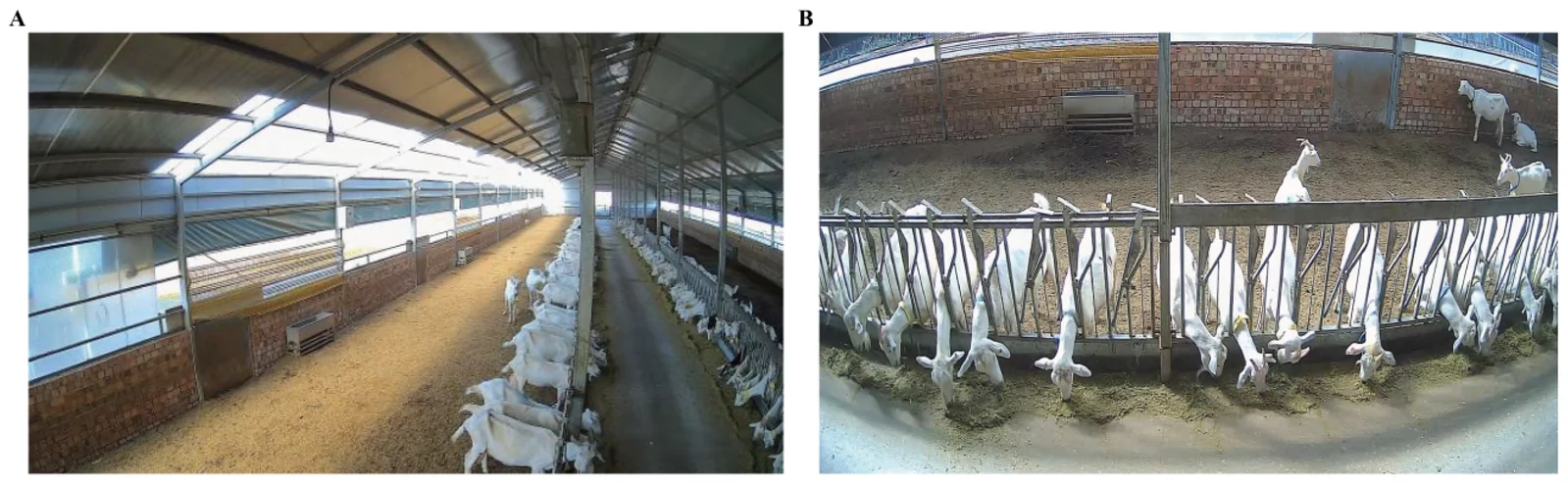
Video-monitoring setup for video-based assessment of feeding behavior in dairy goats. (**A**) Goat counting in the feeding area. (**B**) Detection of individual head-down feeding behavior.

Video acquisition was conducted in two sequential phases rather than simultaneously. In the first phase, group-level feeding-area occupancy was recorded in the five experimental pens. For this purpose, 10 Imou K22-4M-D 2D cameras (Hangzhou Huacheng Network Technology Co., Ltd., Hangzhou, China) were installed at an approximate height of 2.8 m above the feeding lanes, with two cameras assigned to each pen. Each camera covered approximately 27.5 m^2^, and together the two cameras covered the feeding lane within the corresponding pen (Figure 1A). Detection results from the two cameras were analyzed within their respective camera views, and detections from overlapping image regions were not summed directly to obtain the pen-level count. Video recordings were collected continuously for three consecutive days. For each feeding event, the start of feed delivery was defined as the temporal reference point (t = 0), and the first 60 min after feed delivery were extracted for analysis of feeding-area goat counts and their temporal changes.

After the pen-level feeding dynamics had been classified, a second and separate recording phase was conducted for individual head-down feeding behavior. A total of 150 goats were selected from the same five-pen study population, including 75 goats originating from HFA-classified pens and 75 goats from LFA-classified pens. These animals remained in their original pens and were not regrouped for behavioral recording. A total of 12 Imou TB3F-3M cameras (Hangzhou Huacheng Network Technology Co., Ltd., Hangzhou, China) were installed approximately 2.4 m in front of the feeding trough at a height of 1.9 m, with each camera covering approximately 10 m^2^ and oriented toward the head and neck region of the selected goats (Figure 1B). Video recordings were collected during a subsequent three-consecutive-day period, and the first 60 min after each feed delivery were extracted for head-down feeding (HDF) and non-head-down feeding (NHDF). Because the group-level and individual-level recordings were obtained in separate phases and represented different feeding events, frame-level synchronization between the two camera systems was not required. The 150 goats observed in the second phase were drawn from the same original five-pen study population monitored in the first phase, providing a source-pen link between the pen-level feeding classification and the subsequent individual behavioral assessment.

### 2.3. Video Preprocessing, Image Annotation, and Dataset Construction

**Figure 2:**
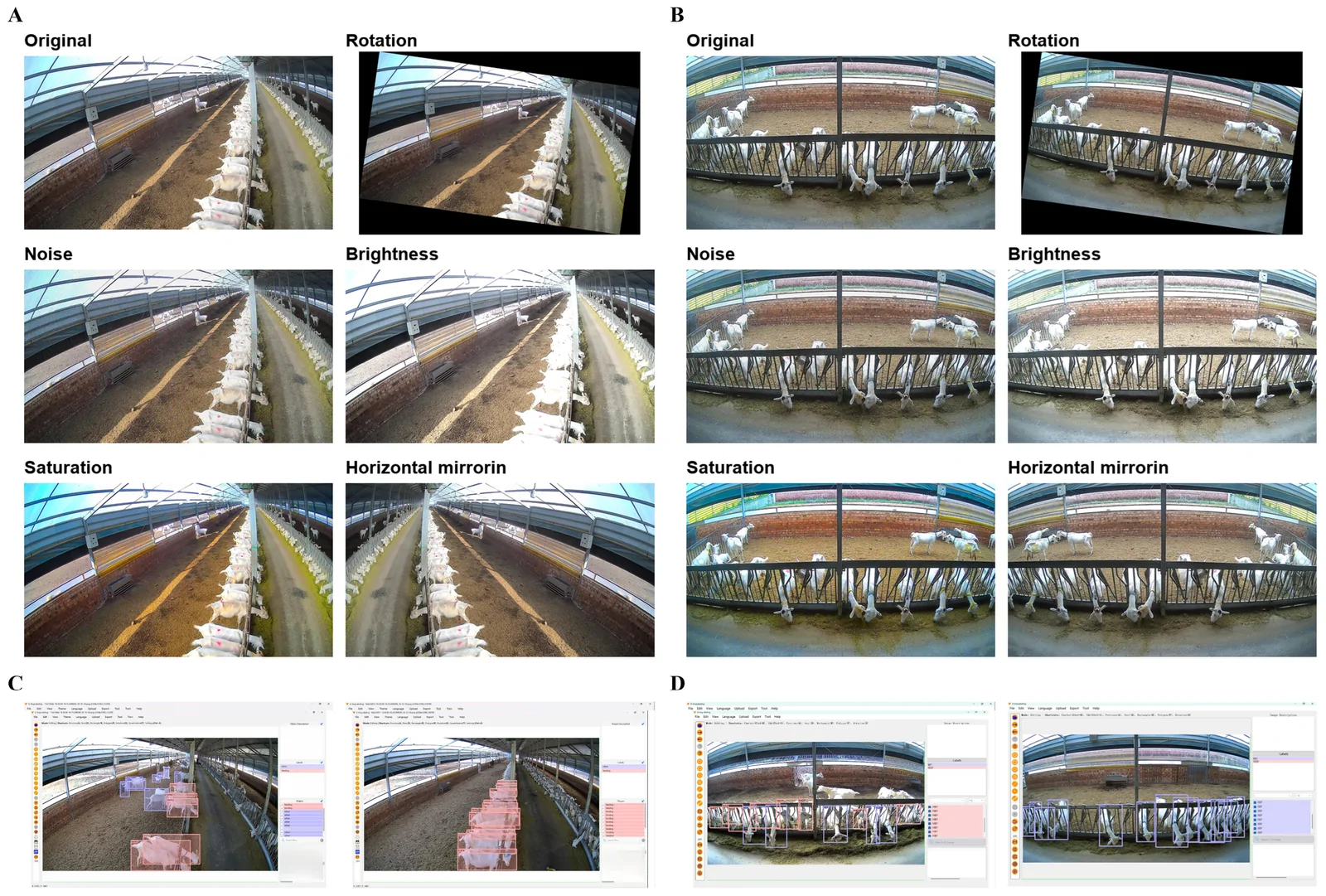
Examples of data augmentation and image annotation. (**A**,**B**) Data augmentation examples for (**A**) feeding-area goat counting and (**B**) individual head-down feeding behavior detection. (**C**,**D**) Image annotation for (**C**) feeding-area goat counting and (**D**) individual head-down feeding behavior detection.

Behavioral quantification used the complete first 60 min after each feed delivery at a sampling rate of 1 frame/s and a resolution of 2304 × 1296 pixels. Separate image subsets were prepared for detector development by sampling candidate frames at approximately 30-s intervals. Images were excluded from the annotation subsets when severe lens obstruction, extreme exposure, substantial motion blur, or animal overlap prevented reliable annotation, or when the feeding area contained no goats. Following screening, 1075 original images were retained for feeding-area goat counting and 1561 for head-posture detection. These numbers represent the manually annotated image subsets rather than the complete video sequences used for behavioral quantification. Examples of the augmentation procedures subsequently applied to the training images are shown in Figure 2A,B.

The original images were manually annotated before augmentation (Figure 2C,D). For feeding-area goat detection, visible goats were annotated with bounding boxes using X-AnyLabeling (v 3.2.6) and classified as “feeding” or “other” according to head position relative to the feeding barrier. For head-posture detection, images were annotated using LabelImg (v 1.8.1). HDF was defined as a visible posture in which the head was lowered toward or into the feed, with the muzzle close to or in contact with the feed; NHDF indicated a visible posture without this configuration. Frames in which head posture could not be determined because of complete occlusion were treated as unobservable and were not assigned to either class. Two trained graduate-student annotators followed the same annotation guidelines. A randomly selected 10% subset was independently annotated by both observers, yielding a mean bounding-box intersection over union (IoU) of 0.87. Disagreements were resolved by a senior member of the research team. A separate intra-observer repeatability assessment was not performed.

Before augmentation, images were partitioned into training, validation, and test sets using recording date and pen identity as the grouping unit, with an approximate target ratio of 8:1:1. All images originating from the same date–pen group were retained within the same partition. Offline augmentation was applied only to the training set. Geometric transformations, including flipping, rotation, and scaling, were accompanied by corresponding bounding-box transformations, whereas photometric transformations did not alter label coordinates. Augmented images were reviewed for annotation quality, and all derivatives of an original image remained within the training set. Online augmentation was likewise restricted to training, whereas validation and test images were not augmented. After augmentation, the feeding-area and head-posture datasets contained 3478 and 3560 images, respectively.

### 2.4. Deep Learning Model Development and Training

Three deep learning-based object detection models, YOLO11, RF-DETR (v1.3), and RT-DETR (v2), were evaluated for goat detection and feeding-area count estimation. YOLO11 was additionally used to construct a binary detection model for individual head-down feeding behavior recognition. YOLO11 incorporates the C3k2 module and C2PSA attention mechanism to improve feature extraction and representation in complex and densely populated scenes (Figure S1A). RF-DETR adopts a DINOv2 Windowed Small backbone and a transformer-based encoder–decoder architecture for multi-scale feature extraction and object detection (Figure S1B). RT-DETR uses an HGNetv2 backbone combined with multi-scale feature fusion and a real-time transformer decoder for end-to-end object detection (Figure S1C). The individual head-down feeding behavior model was developed using YOLO11 with the HDF and NHDF classes defined above. The model was initialized with COCO-pretrained weights and fine-tuned using transfer learning. The input image size was 640 × 640 pixels, and the detection head was configured to predict two classes.

All deep-learning models were trained on a workstation equipped with an NVIDIA GeForce RTX 4090 GPU and 64 GB of system memory. The software environment was based on PyTorch (v2.1). RT-DETR (v2) was implemented using MMDetection 3.0, and RF-DETR (v1.3) was implemented using its official release. A fixed random seed of 42 was used to improve the reproducibility of model training. The principal training settings are summarized in Table 2. The initial learning rates used in the present experiments were 0.01 for RF-DETR and 0.0001 for RT-DETR; the RF-DETR value differed from the default in its official implementation. No exhaustive grid search, random hyperparameter search, or systematic learning-rate sensitivity analysis was performed. Model checkpoints were selected using mean average precision on the validation set, evaluated at an intersection-over-union threshold of 0.5. For each model, the checkpoint achieving the highest validation performance was retained for final evaluation on the held-out test set, and the test set was not used for checkpoint selection.

### 2.5. Quantification of Feeding Behavior Indicators

To quantitatively characterize the temporal dynamics of feeding behavior following feed provision, a series of feeding-related indicators were derived from the frame-by-frame detection results. For each observation time point, the number of goats detected in the feeding area was first calculated from the detection outputs of each camera. Temporal features, including changes in feeding-area goat count, rise and fall times, change rates, peak duration, and leaving frequency, were subsequently derived from the time series of feeding-area goat counts. These indicators were used to characterize the dynamic patterns of goat entry into and departure from the feeding area during the post-feeding period.

(1) Feeding-area goat count (FGC).

FGC was defined as the number of goats detected in the feeding area at each observation time point. The detection results from each camera were used to construct the corresponding time series of feeding-area goat counts. FGC was calculated as follows:

(1) $$X_{t}=\left|\left\{b_{i}\in N_{t}\;|\;{\mathrm{conf}(b}_{i})\geq \tau \right\}\right|$$

where *X_t_* is the number of goats detected in the feeding area at time *t*; *N_t_* is the total number of detection boxes generated by the model at time *t*; *conf*($b_{i}$) is the class confidence score of the *i*th detection box $b_{i}$; and *τ* is the predefined confidence threshold.

(2) Feeding-area goat count change (FGCC).

FGCC was used to quantify the change in the number of goats in the feeding area between two consecutive observations:

(2) $$\Delta X_{t}=X_{t}-X_{t-1}$$

where ${\Delta X}_{t}$ represents the change in feeding-area goat count at time *t*, and *X_t_* and $X_{t-1}$ represent the feeding-area goat counts at the current and preceding time points, respectively.

(3) Rise and fall time of feeding-area goat count (RTFGC and FTFGC).

The rise time of feeding-area goat count (RTFGC) was defined as the interval from the start of feed delivery to the first occurrence of the maximum feeding-area goat count within the 60-min observation window. The fall time of feeding-area goat count (FTFGC) was defined as the interval from the peak time to the time of the subsequent minimum count within the remaining observation window. Both measures were expressed in minutes and calculated as follows:

(3) $$RTFGC=t_{peak}-t_{0}$$

(4) $$FTFGC=t_{min}-t_{peak}$$

where *t*_0_ denotes the start of feed delivery, *t_peak_* denotes the first time at which the maximum feeding-area goat count is reached, and *t_min_* denotes the time of the subsequent minimum count within the same observation window. All time points are expressed in minutes.

(4) Feeding peak duration (FPD).

FPD was defined as the interval from the first occurrence of the maximum feeding-area goat count to the first subsequent time point at which the count began to decline. It was calculated as:

(5) $$FPD=t_{2}-t_{peak}$$

where *t_peak_* is defined above, and *t*_2_ is the first time point at which the feeding-area goat count began to decline after reaching its maximum value (min).

(5) Feeding-area goat count change rate (FGCCR), feeding-area goat count increase rate (FGCIR) and feeding-area goat count decrease rate (FGCDR).

FGCCR represented the net change in the number of goats in the feeding area per unit time. To distinguish the directional dynamics of goats entering and leaving the feeding area, FGCCR was further decomposed into FGCIR and FGCDR. Only changes in the corresponding direction were retained, whereas changes in the opposite direction or no change were assigned a value of zero.

(6) $$v_{t}=\frac{\left|X_{t}-X_{t-1}\right|}{\Delta t}$$

(7) $$v_{t}^{up}=\left\{\begin{array}{c} \frac{X_{t}-X_{t-1}}{\Delta t},X_{t}>X_{t-1}\\ 0,X_{t}\leq X_{t-1} \end{array}\right.$$

(8) $$v_{t}^{down}=\left\{\begin{array}{c} \frac{X_{t}-X_{t-1}}{\Delta t},X_{t}<X_{t-1}\\ 0,X_{t}\geq X_{t-1} \end{array}\right.$$

where *v*_t_ is the feeding-area goat count change rate at time t (goats/min); *v_t_^up^* is the rate of increase in feeding-area goat count (goats/min), representing the positive net change in feeding-area occupancy; *v_t_^down^* is the rate of decrease (goats/min), representing the negative net change in feeding-area occupancy; Δ*t* is the interval between two consecutive observations (min); *X_t_* is the feeding-area goat count at time t; and *X_t−_*_1_ is the feeding-area goat count at the preceding time point.

(6) Frequency of leaving the feeding area (FLFA).

FLFA was defined as the total number of time points at which the feeding-area goat count decreased within a 60-min observation window. It was calculated as:

(9) $$f_{t}^{leave}=\sum_{i=t-59}^{t}I\left(x_{i}<x_{i-1}\right)$$

where *I*(*x_i_* < *x_i_*_−1_) is an indicator function that takes a value of 1 when the condition is satisfied and 0 otherwise.

(7) Individual feeding duration (FD).

FD was calculated based on the proportion of feeding-positive images within each observation interval. For the *i*th time interval, the feeding duration was calculated as:

(10) $$T_{i}=\frac{F_{i}}{60}$$

(11) $$R_{i}=\frac{F_{i}}{S_{i}}\times 100\%$$

where *T_i_* is the feeding duration during the *i*th observation interval (min); *F_i_* is the number of images classified as feeding behavior during the *i*th interval; *S_i_* is the number of valid observable frames for the same goat within the corresponding interval, excluding observations in which head posture could not be determined; *Ri* is the proportion of feeding-positive images during the *i*th interval (%). The resulting detection outputs were subsequently summarized and analyzed to derive feeding-dynamics indicators.

### 2.6. Random Forest (RF) Classification of Pen-Level Feeding Dynamics

Each feature vector was derived from the feeding-area count time series at the pen level. A total of 11 feeding-related features were extracted, including FGC, FGCC, RTFGC, FTFGC, FGCIR, FGCDR, average decrease rates from the peak count to 75%, 50%, and 25% of the peak value (FGCDR75%, FGCDR50%, and FGCDR25%, respectively), FPD, and FLFA. These features characterized pen-level feeding dynamics rather than the behavior of individually identified goats. HFA/LFA reference labels were derived from the temporal characteristics of the feeding-area count curves rather than from an independently established physiological or individual behavioral standard. The RF classifier was therefore used to reproduce these data-derived assignments and to characterize the contribution of individual temporal features. Its performance reflects agreement with the operational labeling scheme rather than independent validation of biologically distinct feeding phenotypes. In subsequent animal-level assessments, HFA/LFA referred to the classification of each goat’s source pen rather than an individual phenotype assigned by the detection model. The RF classifier was implemented using scikit-learn (v1.3), with 200 trees (n_estimators = 200), a maximum tree depth of 10 (max_depth = 10), and random_state = 42. Equal class weights were used (class_weight = None), and the remaining parameters were retained at their default settings. No large-scale grid search or random hyperparameter search was performed.

The contribution of individual feeding-related features to RF classification was first evaluated using Mean Decrease Gini (MDG). Gini impurity was calculated as:

(12) $$\mathrm{Gini}(D)\;=\;1\;-\;\overset{K}{\underset{k=1}{\sum }}p_{k}^{2}$$

where *D* denotes the sample set at a given node, *K* is the number of classes, and $p_{k}$ is the proportion of samples in *D* belonging to class *k*. Lower Gini impurity indicates greater node purity. The MDG value was used to quantify the relative importance of each feature in the RF classification model, with larger values indicating a greater contribution to classification.

To further characterize the contribution and direction of individual features to model predictions, SHapley Additive exPlanations (SHAP) analysis was performed. SHAP values quantify the marginal contribution of each feature to an individual prediction based on the Shapley value from cooperative game theory:

(13) $$\phi_{j}=\sum_{S\subseteq \left\{1,2,\ldots ,p\right\}\setminus \left\{j\right\}}\frac{|S|!(p\;-\;|S|\;-\;1)!}{p!}[f(S\cup \left\{j\})\;-\;f(S)\right]$$

$\phi_{j}$ is the SHAP value of the jth feature, p is the total number of features, and S is a subset of features excluding feature j. f(S) denotes the model output without feature j, whereas f(S∪{j}) denotes the model output after feature j is included.

### 2.7. Individual-Level Head-Down Feeding Behavior Recognition

In the subsequent behavioral observation phase, a YOLO11-based object detection model was applied to the 150 selected goats, comprising 75 animals originating from HFA-classified pens and 75 from LFA-classified pens. The purpose of this analysis was to compare individual feeding-posture observations between goats originating from the two pen-level feeding-activity categories, rather than to assign or validate individual HFA/LFA labels using the group-level model. For behavioral quantification, only observations in which head posture could be clearly determined were included. Frames in which the head was fully obscured and posture could not be determined were recorded as unobservable and excluded from the denominator. HDF and NHDF proportions were therefore calculated relative to valid observable time, while unobservable observations were summarized separately. The model was initialized using COCO-pretrained weights and fine-tuned through transfer learning. Input images were resized to 640 × 640 pixels, and the detection head was configured to predict two classes. The performance of the object detection models was evaluated through precision (*P*), recall (*R*), average precision (*AP*), *mAP*, *F*1-score (*F*1), and accuracy (*Acc*). *mAP* was reported both at an IoU threshold of 0.5 (*mAP*@0.5) and as the mean across IoU thresholds from 0.50 to 0.95 (*mAP*@0.5:0.95).

These metrics were calculated as follows:

(14) $$P=\frac{TP}{TP+FP}\times 100\%$$

(15) $$R=\frac{TP}{TP+FN}\times 100\%$$

(16) $$mAP=\frac{1}{c}\sum_{i=1}^{c}APi\times 100\%$$

(17) $$F1=\frac{2\times P*R}{P+R}$$

(18) $$Acc=\frac{\mathrm{TP}+\mathrm{TN}}{\mathrm{TP}+\mathrm{TN}+\mathrm{FP}+\mathrm{FN}}\times 100\%$$

where *TP*, *FP*, and *FN* denote true positives, false positives, and false negatives, respectively; c is the total number of classes; *i* is the index of the ith class; and *APi* is the average precision for the *i*th class.

For the object detection models, the training objective was jointly optimized using classification loss (cross-entropy loss), bounding-box regression loss (L1 loss), and generalized intersection-over-union (GIoU) loss, as applicable to the corresponding model architecture. For the RF classification model, the receiver operating characteristic (ROC) curve and the area under the curve (AUC) were used to assess the discriminative ability of the RF model. A macro-average strategy was used to calculate the overall AUC. The ROC curve was constructed using the false-positive rate (FPR) and true-positive rate (TPR), where FPR represents the proportion of LFA samples incorrectly classified as HFA and TPR represents the proportion of HFA samples correctly classified as HFA. AUC ranges from 0 to 1, with higher values indicating better discriminative performance. The corresponding metrics were calculated as follows:

(19) $$\mathrm{FPR}=\frac{\mathrm{FP}}{\mathrm{FP}+\mathrm{TN}}$$

(20) $$\mathrm{TPR}=\frac{\mathrm{TP}}{\mathrm{TP}+\mathrm{FN}}$$

A confusion matrix was used to visualize the classification results of the RF model. The diagonal elements represent correctly classified samples, whereas the off-diagonal elements represent misclassified samples, allowing the error patterns between HFA and LFA groups to be further assessed.

### 2.8. Exploratory Assessment of Production Performance and Physiological Indicators in HFA and LFA Pens

For the exploratory production and physiological assessment, 24 goats were selected from the 150 animals included in the subsequent behavioral observation phase, comprising 12 goats originating from HFA-classified pens (mean head-down feeding duration: 32.56 ± 1.00 min) and 12 from LFA-classified pens (29.51 ± 1.00 min). Selection took account of source-pen classification and observed HDF duration. The selected goats had BCS of 2.75–3.00 and were at a similar stage of lactation (120 ± 10 days in milk). Animals with apparent health abnormalities or incomplete production records were excluded to reduce potential confounding. All selected goats remained in their original pens, with no regrouping or changes in group composition. Personnel responsible for animal selection were aware of the source-pen classifications because these classifications formed part of the sampling procedure. In contrast, subsequent milk-yield recording, blood collection, and serum-indicator measurements were performed by personnel blinded to the HFA/LFA source-pen classification. Consequently, the production and physiological comparisons were restricted to apparently healthy animals and were intended as exploratory assessments rather than validation of disease-detection or health-monitoring performance.

Feed intake was assessed at the pen level rather than individually. For each measurement day, the amount of feed offered to each pen was recorded, and refusals were weighed before the following morning feeding. Pen-level dry matter intake (DMI) was calculated from the difference between feed offered and refusals on a dry-matter basis, divided by the number of goats housed in the pen and averaged across the three-day measurement period. Thus, the reported DMI represents an estimated pen-average daily DMI per goat and should not be interpreted as an individually measured DMI for the 150 behaviorally observed goats or the 24-goat physiological subset. Milk yield, in contrast, was recorded individually for each of the 24 selected goats during the three-day measurement period. Milk samples were collected during the morning and evening milking sessions at a ratio of 6:4 and thoroughly mixed to obtain a representative daily sample. Approximately 50 mL of milk was collected from each goat and transferred into centrifuge tubes pretreated with preservative. Samples were stored at 4 °C until analysis, and milk composition was determined using a LACTOSCAN MCCW V1 rapid milk analyzer (MILKOTRONIC Ltd., Nova Zagora, Bulgaria).

At the end of the experimental period, approximately 20 mL of blood was collected from the jugular vein of each selected goat using vacuum blood collection tubes. Blood samples were allowed to stand at room temperature for 30 min and then centrifuged at 4000× *g* for 10 min. The resulting serum was carefully collected and stored at −80 °C until further analysis. Serum immune, antioxidant, and neuroendocrine-related indicators were determined using commercial enzyme-linked immunosorbent assay (ELISA) kits according to the manufacturer’s instructions. The immune-related indicators included immunoglobulin A (IgA), immunoglobulin G (IgG), immunoglobulin M (IgM), interleukin-2 (IL-2), interleukin-10 (IL-10), and tumor necrosis factor-alpha (TNF-α). The antioxidant indicators included superoxide dismutase (SOD), catalase (CAT), glutathione peroxidase (GSH-Px), total antioxidant capacity (T-AOC), and malondialdehyde (MDA). The neuroendocrine-related indicators included serotonin (5-HT), γ-aminobutyric acid (GABA), cortisol (COR), brain-derived neurotrophic factor (BDNF), norepinephrine (NE), neuropeptide Y (NPY), leptin (LEP), and corticotropin-releasing hormone (CRH). All ELISA kits were purchased from Wuhan GeneMei Biotechnology Co., Ltd. (Wuhan, China). Because blood samples were collected only once at the end of the experimental period, the serum analyses were treated as cross-sectional exploratory comparisons and were not intended to characterize temporal physiological dynamics or infer causal relationships between feeding activity and physiological status.

### 2.9. Statistical Analysis

Continuous variables were assessed for normality before statistical analysis. Differences between the HFA and LFA groups were evaluated using an independent-samples *t*-test for normally distributed data and the Mann–Whitney U test for non-normally distributed data. For the independent-samples *t*-test, homogeneity of variance was assessed before analysis, and the appropriate test was selected according to the variance assumption. A two-sided *p* < 0.05 was considered statistically significant. Data processing, statistical analysis, and visualization were performed operating Microsoft Excel (v 2024; Microsoft Corporation, Redmond, WA, USA), SPSS Statistics (v 24.0; IBM Corp., Armonk, NY, USA), R software (v 4.2.3; R Foundation for Statistical Computing, Vienna, Austria), and Origin (v 2024;OriginLb Corporation, Northampton, MA, USA).

## 3. Results

### 3.1. RF-DETR Outperformed Other Deep Learning Models for Feeding-Area Goat Counting

**Figure 3:**
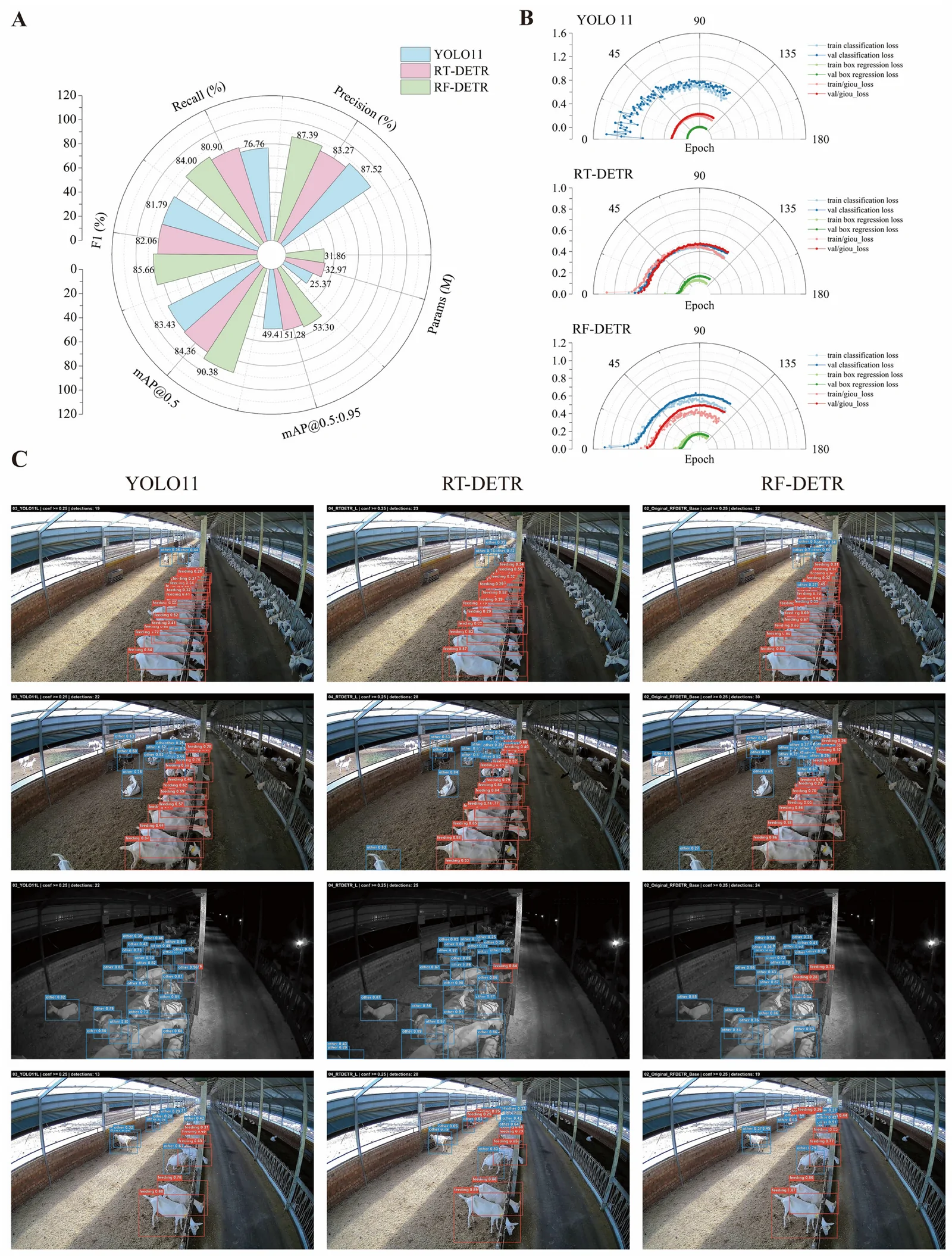
Performance comparison of YOLO11, RF-DETR, and RT-DETR for feeding-area goat detection. (**A**) Quantitative performance evaluation. (**B**) Training and validation loss curves. (**C**) Representative detection results.

This study first aimed to characterize group-level feeding dynamics. Overall, RF-DETR achieved the best overall balance of detection accuracy and model performance among the three models. It achieved an *F*1-score, *mAP*@0.5, and *mAP*@0.5:0.95 of 85.66%, 90.38%, and 53.30%, respectively, with a well-balanced *P* (87.39%) and *R* (84.00%). In comparison, YOLO11 had the smallest number of parameters (25.37 M), but showed the lowest *R* (76.76%), indicating a relatively higher rate of missed detections. RT-DETR showed intermediate performance across most evaluation metrics but had the largest model size (32.97 M) (Figure 3A).

The training loss curves indicated that all three models converged effectively. YOLO11 showed relatively stable generalization in classification loss, although its bounding-box regression and GIoU losses reached a plateau at an earlier stage. RT-DETR exhibited a rapid decrease in loss during the first 10 epochs, followed by a gradual stabilization. RF-DETR converged rapidly during the early training stage and remained stable thereafter, with relatively consistent bounding-box regression performance between the training and validation sets (Figure 3B). Qualitative inspection further showed that all three models were capable of detecting dairy goats in the feeding area under the study conditions (Figure 3C). Considering the quantitative evaluation metrics, convergence characteristics, and qualitative detection results, RF-DETR was selected for subsequent feeding-dynamics analysis based on its detection performance under the evaluated conditions.

### 3.2. Dairy Goats Exhibited Distinct Temporal Dynamics of Feeding-Area Occupancy After Feed Delivery

**Figure 4:**
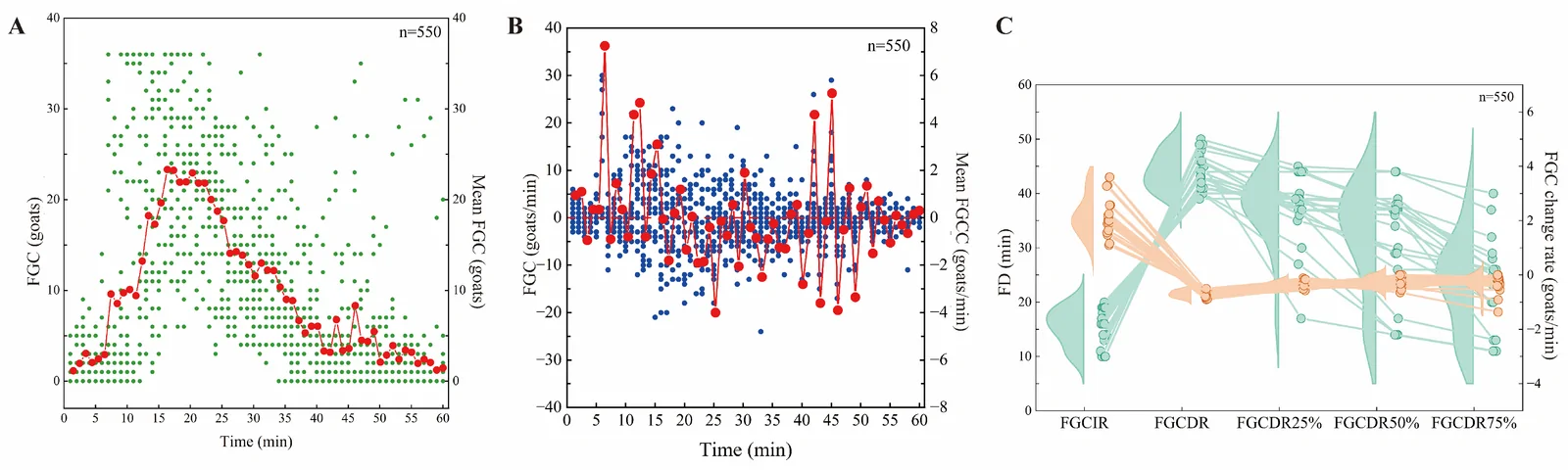
Temporal dynamics of feeding-area goat counts and feeding behavior after feed provision. (**A**) Changes in feeding-area goat count (FGC) during the 60-min observation period after feed provision. Green dots represent observed FGC values, while the red line represents the mean FGC at each time point. (**B**) Feeding-area goat count change (FGCC) during the 60-min observation period. Blue dots represent observed FGCC values, while the red line represents the mean FGCC at each time point. (**C**) Distribution and individual variation of feeding duration (FD) and feeding-area goat count change rates (FGCCR), including increase rate (FGCIR), decrease rate (FGCDR), and decrease rates at 25%, 50%, and 75% of the peak FGC (FGCDR25%, FGCDR50%, and FGCDR75%, respectively). Green distributions and points represent FD (left y-axis), whereas orange distributions and points represent feeding-area goat count change rates (right y-axis).

Based on the feeding-area goat counts derived from the detection results, dairy goats exhibited pronounced temporal dynamics in feeding-area occupancy after feed delivery. Within the first 60 min after feed delivery, FGC increased rapidly after feed delivery and reached its maximum at 16 min, followed by a relatively stable peak period of approximately 6 min before gradually declining. By 30 and 50 min after feed delivery, FGC had decreased to approximately 50% and 25% of the peak value, respectively, indicating that the intensity of group-level feeding activity varied substantially across the post-feeding period (Figure 4A). FGCC further characterized the dynamics of goats entering and leaving the feeding area. During the first 1–15 min after feed delivery, FGCC was predominantly positive, with positive changes accounting for more than 85% of the observations, indicating a sustained influx of goats into the feeding area. A distinct transition from predominantly positive to negative FGCC occurred between 15 and 16 min, coinciding with the time at which FGC reached its maximum. From 16 to 40 min, FGCC was predominantly negative, indicating a gradual departure of goats from the feeding area. During 41–60 min, FGCC progressively approached zero, suggesting that the number of goats remaining in the feeding area became relatively stable as overall feeding activity gradually diminished (Figure 4B). Notably, the temporal profiles of group-level feeding behavior were not characterized by a uniform linear decline but instead exhibited distinct temporal heterogeneity, with two contrasting feeding patterns identified. One pattern was characterized by a “rapid-rise and slow-decline” profile, with a higher FGCIR (3.40 goats/min) and a slower decline (FGCDR = −0.51 goats/min). In contrast, the other pattern exhibited a “slow-rise and rapid-decline” profile, characterized by a lower FGCIR (1.89 goats/min) and a faster decline (FGCDR = −0.84 goats/min) (Figure 4C). Collectively, dairy goats generally exhibited a “rapid entry–peak aggregation–gradual departure” feeding response following feed delivery, while substantial variation existed in the rate and duration of these phases. This temporal heterogeneity provided a basis for subsequently characterizing individual differences in feeding activity.

### 3.3. Group-Level Feeding Dynamics Distinguished HFA and LFA Pens

**Figure 5:**
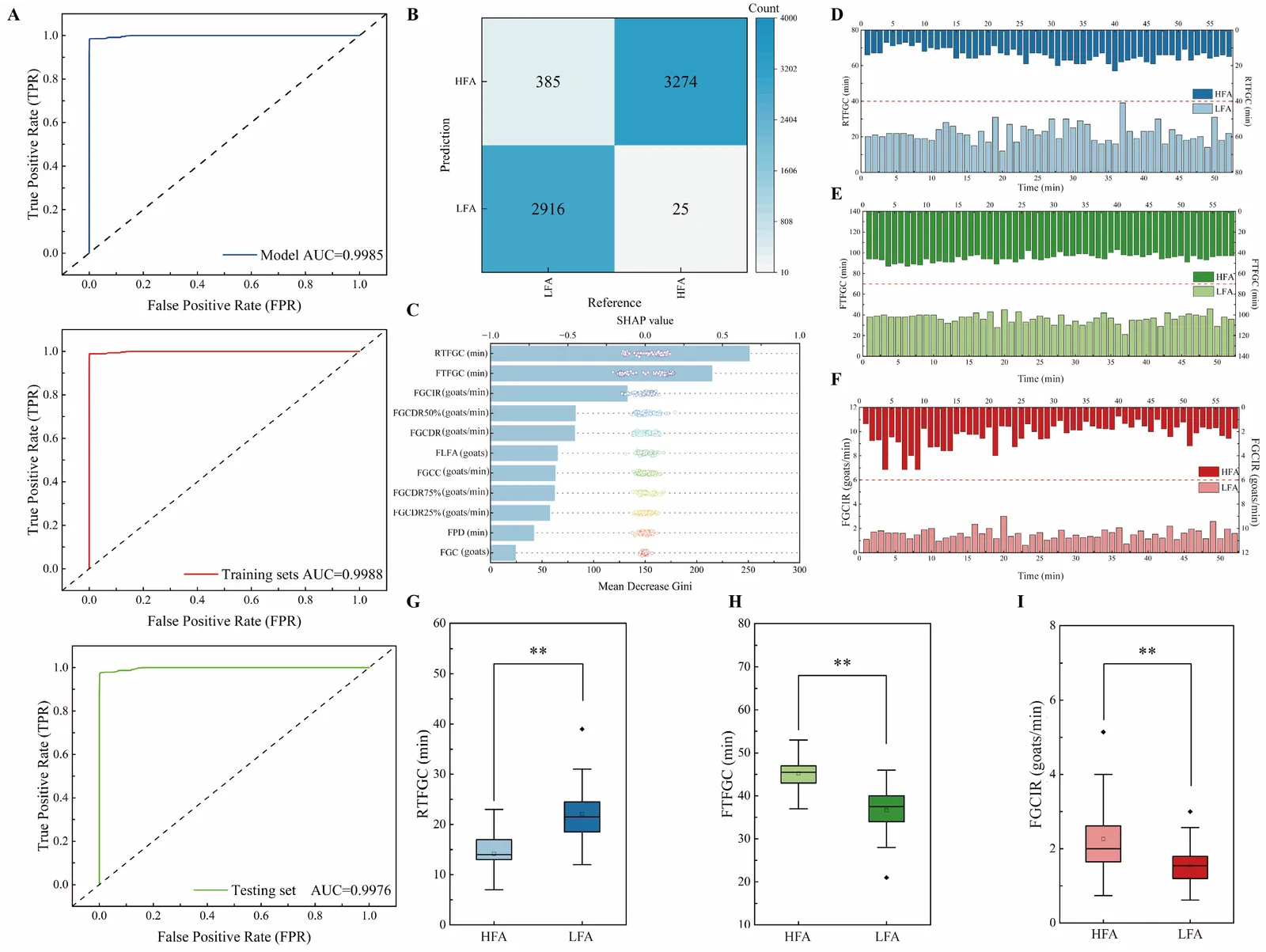
Random forest (RF) classification performance, feature importance, and key feeding-dynamics differences between high feeding activity (HFA) and low feeding activity (LFA) goats. (**A**) Area under the curve (AUC) of the RF model. (**B**) Confusion matrix of the RF classification results (*n* = 6600 records). (**C**) Feature importance ranking based on mean decrease in Gini impurity and SHAP analysis. (**D**–**I**) Comparisons of the three most important feeding-dynamics features between HFA and LFA goats: (**D**,**G**) RTFGC, (**E**,**H**) FTFGC, and (**F**,**I**) FGCIR. Note: RTFGC, rise time of feeding-area goat count, defined as the interval from feed provision to the first occurrence of the maximum feeding-area goat count; FTFGC, fall time of feeding-area goat count, defined as the interval from the peak to the subsequent minimum within the observation window; FGCIR, feeding-area goat count increase rate; FGCC, feeding-area goat count change; FGCDR, feeding-area goat count decrease rate; FGCDR25%, average decrease rate from the peak feeding-area goat count to 25% of the peak; FGCDR50%, average decrease rate from the peak to 50% of the peak; FGCDR75%, average decrease rate from the peak to 75% of the peak; FLFA, frequency of leaving the feeding area; FPD, feeding peak duration; FGC, feeding-area goat count. ** *p* < 0.01.

In total, 11 temporal features derived from pen-level feeding-area count series were used in the RF analysis to distinguish HFA and LFA feeding patterns. The RF classifier showed consistent performance between the training and testing sets, with AUC values of 0.9985 for the overall cohort, 0.9988 for the training set, and 0.9976 for the testing set (Figure 5A). Overall, the model achieved an *Acc* of 93.79%, *P* of 94.32%, and *F*1-score of 93.77%, with classification performance evaluated against the operational HFA/LFA record labels (Table 3). The RF results were interpreted as agreement with the data-derived reference labels rather than evidence of independently validated individual feeding phenotypes. The confusion matrix for the overall cohort showed that 410 of 6600 samples were misclassified, corresponding to an overall misclassification rate of 6.21%. Among these misclassified samples, 385 true LFA samples were incorrectly classified as HFA, whereas only 25 true HFA samples were misclassified as LFA (Figure 5B). Additionally, Feature importance analysis based on mean decrease in Gini impurity and SHAP values identified RTFGC (251.45), FTFGC (215.19), and FGCIR (132.94) as the key features discriminating HFA from LFA goats. Among these features, FTFGC showed the largest mean absolute SHAP value (Mean |SHAP| = 0.135), indicating that the duration of the declining phase contributed most strongly to the RF classification. Longer FTFGC values represented longer intervals from peak occupancy to the subsequent minimum within the observation window. Similarly, shorter RTFGC and higher FGCIR were associated with earlier peak occupancy and a more rapid increase in feeding-area goat count. In comparison, FGCDR50%, FGCDR, and FLFA showed intermediate importance, whereas FPD and FGC contributed relatively little to classification (Figure 5C). These findings suggest that the temporal dynamics of feeding-area goat numbers, particularly the timing and rate of changes in goat presence, were more informative for distinguishing HFA from LFA goats than static measures of feeding-area occupancy, which showed relatively limited discriminatory value. Based on these feature-importance results, the distributions of the three key temporal features were further compared between HFA and LFA goats. The RTFGC of LFA goats was 22.10 ± 5.14 min, significantly longer than that of HFA goats (14.16 ± 3.47 min, *p* < 0.01), representing a 56.09% increase (Figure 5D,G). Conversely, FTFGC was significantly shorter in LFA goats (36.65 ± 4.72 min) than in HFA goats (45.24 ± 3.61 min, *p* < 0.01), with the HFA group showing a 23.43% longer declining phase (Figure 5E,H). In addition, FGCIR was significantly lower in LFA goats (1.54 ± 0.44 goats/min) than in HFA goats (2.27 ± 1.01 goats/min, *p* < 0.01), corresponding to a 47.03% higher increase rate in the HFA group (Figure 5F,I). These results suggest that HFA goats exhibited faster feeding initiation and a more sustained feeding response, whereas LFA goats showed slower initiation and a shorter duration of feeding activity.

### 3.4. Individual-Level Feeding Behavior Supported Higher Feeding Activity in HFA Goats

**Figure 6:**
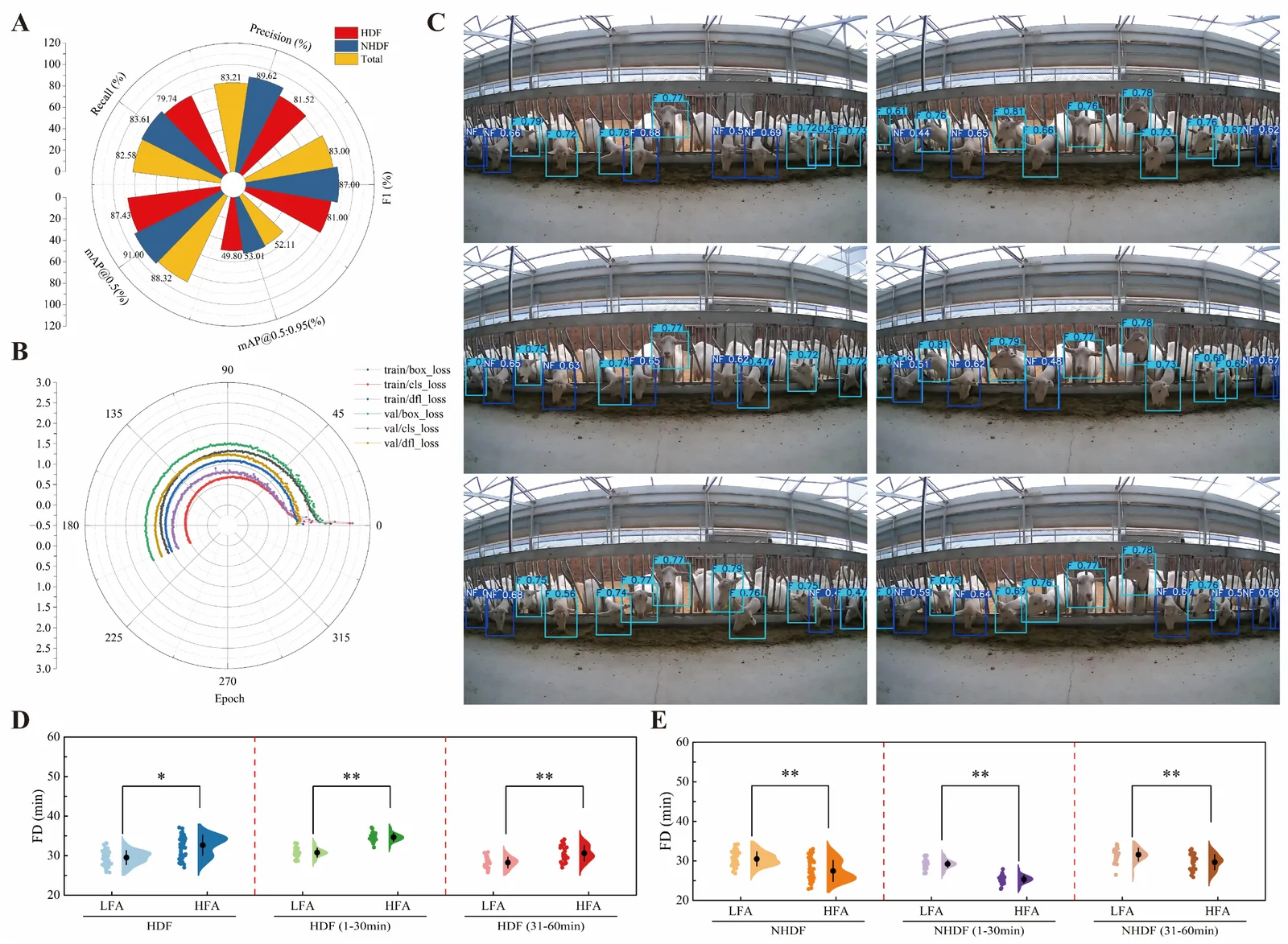
Individual-level detection of feeding behavior and differences in head-down feeding between high feeding activity (HFA) and low feeding activity (LFA) goats. (**A**) Quantitative performance evaluation of the YOLO11-based individual feeding behavior detection model. (**B**) Training and validation loss curves. (**C**) Representative detection results; (**D**,**E**) Comparison of the proportions of head-down feeding (HDF) and non-head-down feeding (NHDF) among valid observable records from goats originating from HFA and LFA pens. * *p* < 0.05 and ** *p* < 0.01.

In the subsequent recording phase, individual HDF/NHDF observations were compared between 75 goats from HFA pens and 75 goats from LFA pens. The model automatically classified head posture into two behavioral states: HDF and NHDF. The overall *P*, *R*, *mAP*@0.5, *mAP*@0.5:0.95, and *F*1-score were 83.21%, 82.58%, 88.32%, 52.11%, and 83.00%, respectively. For NHDF, the *P*, *R*, and *F*1-score were 89.62%, 83.61%, and 87.00%, respectively, whereas the corresponding values for HDF were 81.52%, 79.74%, and 81.00%. All class-specific *F*1-scores exceeded 80%, and *mAP*@0.5 values exceeded 85%. The model converged rapidly during the first 50 epochs, and the losses became relatively stable after approximately 180 epochs (Figure 6A–C). The individual-level behavioral analysis further revealed clear differences between HFA and LFA. Overall, the proportion of HDF was significantly higher in HFA goats than in LFA goats (*p* < 0.05), whereas the proportion of NHDF was significantly lower (*p* < 0.01). Based on the temporal pattern of group-level feeding dynamics, the 60-min observation period was divided into an early post-feeding period (1–30 min) and a late post-feeding period (31–60 min). During 1–30 min after feeding, HFA goats exhibited a significantly higher proportion of HDF and a significantly lower proportion of NHDF than LFA goats (both *p* < 0.01). These differences remained significant during 31–60 min, with HFA goats maintaining a higher HDF proportion and a lower NHDF proportion than LFA goats (both *p* < 0.01) (Figure 6D–E). These findings provide individual-level behavioral evidence supporting the group-level classification, indicating that HFA goats not only exhibited more rapid and sustained changes in feeding-area occupancy but also spent a greater proportion of time in the head-down feeding state.

### 3.5. Higher Feeding Activity Was Associated with Greater Feed Intake and Milk Yield

**Figure 7:**
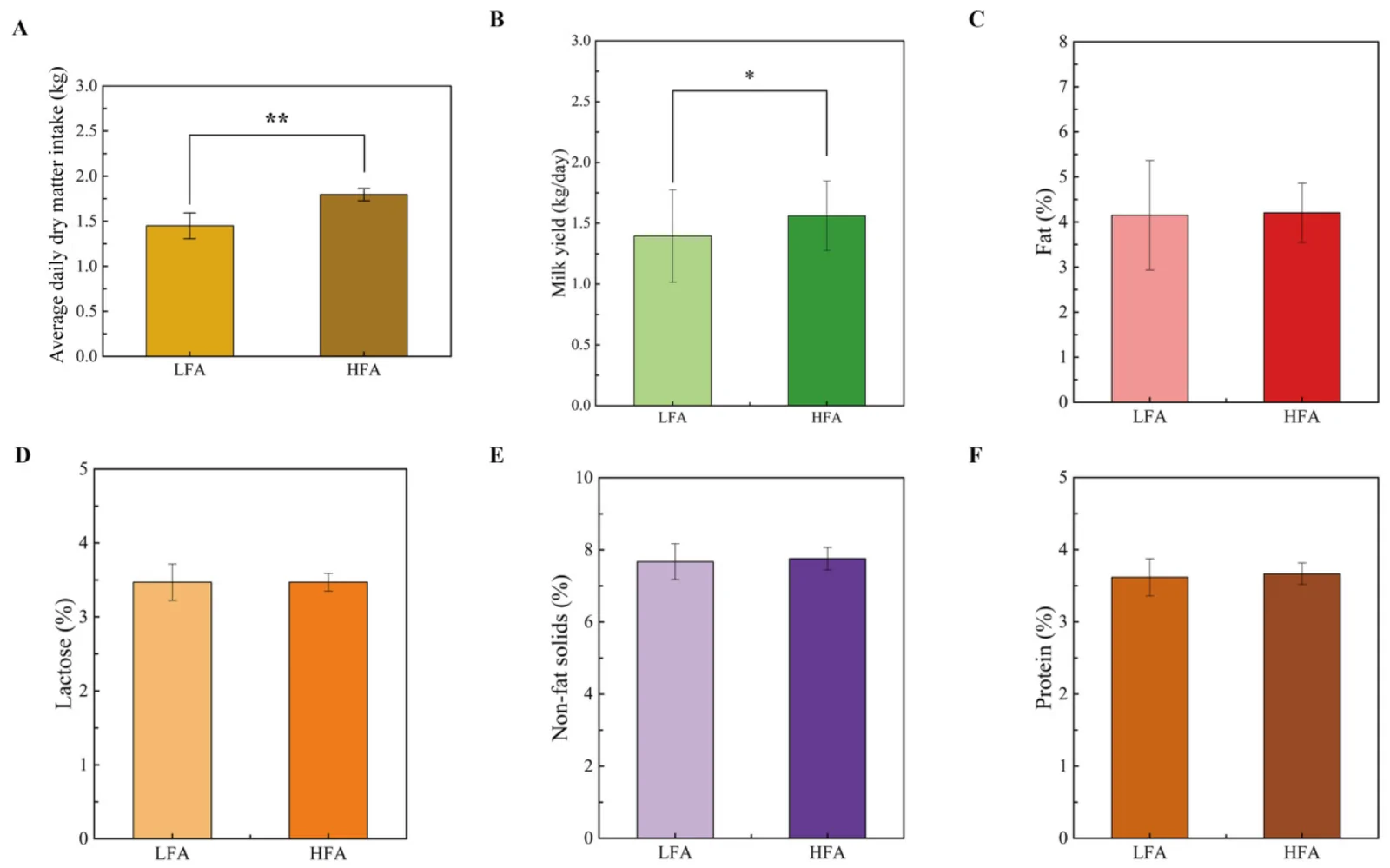
Feed intake and milk production performance of high feeding activity (HFA) and low feeding activity (LFA) goats. (**A**) Estimated pen-average daily dry matter intake (DMI) per goat, calculated from pen-level feed and refusal measurements. (**B**–**F**) Individual milk-production and composition measurements in the selected 24-goat subset. * *p* < 0.05 and ** *p* < 0.01.

Pen-average DMI was summarized by source-pen category, whereas milk yield and composition were assessed in the selected 24-goat subset. At the group level, average DMI was significantly higher in HFA goats than in LFA goats (*p* < 0.01), indicating an association between the feeding-activity classification and feed intake within the selected 24-goat subset (Figure 7A). Consistent with the difference in feed intake, HFA goats also exhibited significantly higher milk yield than LFA goats (*p* < 0.05) among the 24 goats included in the exploratory production assessment (Figure 7B). In contrast, no significant differences were observed between HFA and LFA goats in milk fat, lactose, non-fat solids, or protein contents (*p* > 0.05) (Figure 7C–F). These findings indicate that higher feeding activity was associated with greater feed consumption and milk production, while the classification was not accompanied by detectable differences in the major components of milk.

### 3.6. Exploratory Comparisons of Selected Serum Physiological Indicators in Goats from HFA and LFA Pens

**Figure 8:**
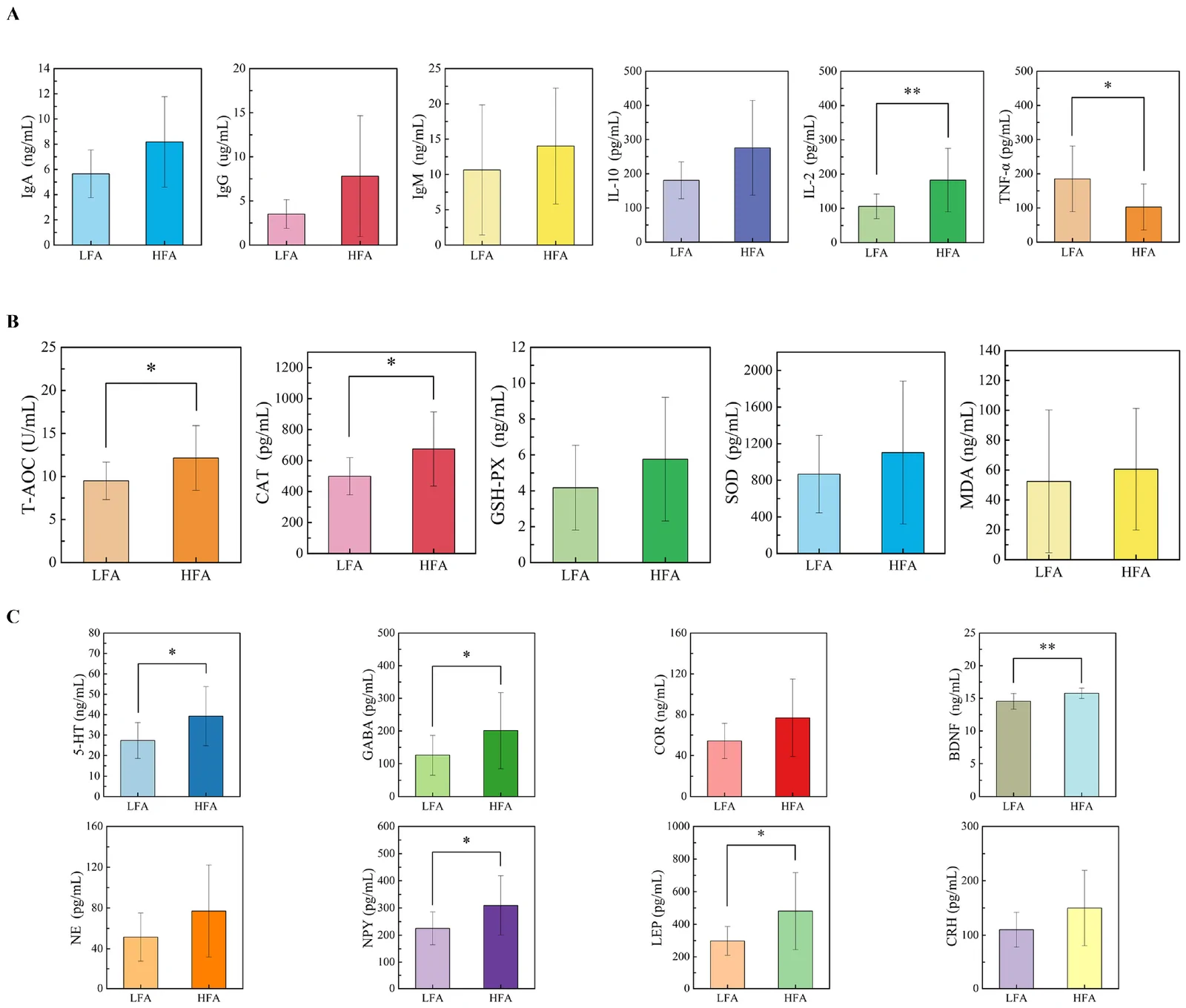
Selected serum physiological indicators in goats originating from high feeding activity (HFA) and low feeding activity (LFA) pens. (**A**) Serum immune-related indicators. (**B**) Serum antioxidant-related indicators. (**C**) Serum neuroendocrine-related indicators. * *p* < 0.05, ** *p* < 0.01. Note: The immune-related indicators included immunoglobulin A (IgA), immunoglobulin G (IgG), immunoglobulin M (IgM), interleukin-2 (IL-2), interleukin-10 (IL-10), and tumor necrosis factor-alpha (TNF-α). The antioxidant-related indicators included superoxide dismutase (SOD), catalase (CAT), glutathione peroxidase (GSH-Px), total antioxidant capacity (T-AOC), and malondialdehyde (MDA). The neuroendocrine-related indicators included serotonin (5-HT), γ-aminobutyric acid (GABA), cortisol (COR), brain-derived neurotrophic factor (BDNF), norepinephrine (NE), neuropeptide Y (NPY), leptin (LEP), and corticotropin-releasing hormone (CRH).

As an exploratory cross-sectional analysis, selected serum immune-, antioxidant-, and neuroendocrine-related indicators were compared between HFA and LFA goats. For immune-related indicators, serum IgA, IgM, IgG, and IL-10 concentrations were numerically higher in HFA goats than in LFA goats, although these differences did not reach statistical significance (*p* > 0.05). In contrast, the concentration of IL-2 was significantly higher in HFA goats than in LFA goats (*p* < 0.01), while serum TNF-α concentration was significantly higher in LFA goats than in HFA goats (*p* < 0.05) (Figure 8A). For antioxidant-related indicators, HFA goats exhibited significantly higher serum CAT and T-AOC concentrations than LFA goats (*p* < 0.05). Although serum SOD, GSH-Px, and MDA concentrations were also numerically higher in HFA goats, the differences were not statistically significant (*p* > 0.05) (Figure 8B). Notably, several neuroendocrine-related indicators also differed between the two groups. Serum concentrations of 5-HT, GABA, NPY, and LEP were significantly higher in HFA goats than in LFA goats (*p* < 0.05), while BDNF showed an even greater increase in the HFA group (*p* < 0.01). In contrast, no significant differences were observed in COR, NE, or CRH between HFA and LFA goats (*p* > 0.05) (Figure 8C). Collectively, these cross-sectional comparisons identified differences in several selected serum indicators between HFA and LFA goats. Given the limited sample size, these findings should be regarded as exploratory and require confirmation in larger independent cohorts.

## 4. Discussion

### 4.1. Computer Vision for Automated Feeding-Behavior Monitoring

Feeding behavior is an important behavioral phenotype reflecting nutrient intake, health status, and production performance in ruminants, while deviations in feeding behavior may serve as early indicators of deteriorating health [20]. For example, dairy goats with prolonged feeding duration but reduced feeding frequency are more likely to exhibit lower rumen pH, suggesting an increased risk of subacute ruminal acidosis [21]. However, conventional approaches based on direct observation, video recording, or contact-based sensors are often labor-intensive and difficult to apply for continuous, objective, and individual-level monitoring under commercial farming conditions [14]. This challenge is further amplified in group-housed dairy goats, where feeding behavior is highly dynamic and heterogeneous across individuals rather than being adequately described by simple feeding/non-feeding states [20]. In the present study, we developed a computer vision- and machine learning-based framework to characterize feeding dynamics as a quantitative digital behavioral phenotype. By emphasizing temporal changes in feeding-area occupancy rather than feeding presence alone, this framework offers a way to characterize behavioral variation under commercial-farm conditions. The production and serum comparisons provide exploratory context for the potential relevance of these behavioral measures, rather than confirmatory biological validation.

Computer vision provides a non-invasive approach for characterizing feeding behavior, particularly under commercial farming conditions where large group size, frequent animal–animal occlusion, variable postures, and changing illumination can substantially complicate automated detection [22]. In the present study, YOLO11, RF-DETR, and RT-DETR all achieved satisfactory goat detection performance, whereas RF-DETR showed the most favorable overall balance across *P*, *R*, *F*1-score, and *mAP* and was therefore selected for subsequent feeding-dynamics analysis. This superior performance may be related to the Transformer-based architecture and DINOv2 backbone of RF-DETR, which facilitate the representation of contextual information and the detection of multiple closely distributed animals in complex scenes [23]. Importantly, the value of the present framework extends beyond model accuracy itself. Previous research has demonstrated the feasibility of sensor-based monitoring of feeding and ruminating behavior in sheep and goats [24], but wearable or contact-based sensors may increase equipment maintenance requirements and potentially interfere with normal animal behavior [25]. By contrast, our approach uses non-contact video acquisition and converts continuous goat detections into quantitative time-series features, thereby extending computer vision from simple animal detection to the characterization of feeding dynamics. From a practical perspective, these video-derived measures could complement routine observations by summarizing the timing and rate of post-feeding occupancy changes. Their potential value for feeding-management decisions still requires evaluation under routine farm operation. Model selection also involves a trade-off between detection performance and deployment requirements. RF-DETR was selected for the subsequent feeding-dynamics analysis based on its performance under the evaluated conditions, whereas the YOLO11 configuration had fewer parameters. This makes YOLO11 a candidate for resource-constrained deployment, although parameter count alone does not establish inference speed, runtime memory use, or energy consumption. These deployment characteristics were not evaluated on edge devices in the present study. Future implementation should therefore assess them on the intended hardware alongside detection performance and the accuracy of the derived occupancy measures.

### 4.2. Interpretation of Feeding Dynamics and Head-Down Feeding Behavior

Beyond detection accuracy, continuous video observations revealed a distinct temporal pattern of feeding-area occupancy following feed delivery. Dairy goats generally exhibited a “rapid entry–peak aggregation–gradual departure” response. This pattern is consistent with previous observations that small ruminants rapidly increase feeding activity after feed delivery and that early post-feeding intake is closely related to daily feed intake [3,26,27]. Importantly, the feeding response was not adequately described by a single time-point measure of feeding-area occupancy. Differences in FGCIR and FGCDR revealed distinct feeding trajectories, ranging from a rapid increase in feeding-area occupancy followed by a gradual decline to a slower increase followed by a more rapid decline. These findings highlight substantial heterogeneity in the temporal organization of feeding behavior among dairy goats even under standardized feeding conditions [20]. Such variation may reflect not only feeding motivation but also body weight, social rank, and access to feeding space. Body weight has been associated with dominance rank in goats [28], while research in dairy goats has shown that feeding-space restriction and social rank influence feeding and queuing behavior [2]. Selective feeding can also alter the composition of the remaining mixed ration [29]. If preferred nutrient-rich fractions are depleted earlier, animals gaining access later may encounter a less favorable ration. Delayed or reduced occupancy may therefore reflect restricted feeding opportunities rather than lower intrinsic feeding motivation. These remain possible explanations rather than mechanisms demonstrated in the present study.

Building on these distinct feeding trajectories, RTFGC, FTFGC, and FGCIR emerged as the most informative features for distinguishing HFA from LFA goats. Together, these variables capture complementary aspects of feeding dynamics, including the timing of peak occupancy, the rate of increase in feeding-area occupancy, and the persistence of the feeding response, highlighting the importance of temporal patterns rather than static measures of goat presence [3]. This dynamic pattern is consistent with previous studies showing substantial inter-individual variation in feeding behavior in dairy goats and associations between early post-feeding behavior and ruminal responses [26,30]. The relatively rapid and sustained feeding response observed in HFA goats also resembles the “big loader” feeding strategy described in dairy goats, whereas the less pronounced and shorter feeding response of LFA goats may be more consistent with a “nibbler” strategy [30]. Importantly, previous work in dairy cattle has shown that changes in feeding frequency, rate, and duration can precede or accompany health disorders, supporting the potential of feeding dynamics as an early behavioral indicator of altered animal status [27]. Thus, the temporal features identified here may provide useful digital phenotypes for longitudinal assessment of individual feeding behavior and precision livestock management [30]. Furthermore, the reported RF error pattern was asymmetric, with LFA-labeled records more frequently assigned to HFA than vice versa. This indicates that some lower-activity patterns may be overlooked when reproducing the operational grouping. Overlap between the temporal characteristics of the two groups is a possible explanation, but this was not directly evaluated. These errors should be considered before using the classifier to flag lower-activity events; they do not establish sensitivity for detecting low intake or disease in individual animals.

Because HFA/LFA classification concerned pen-level dynamics, subsequent HDF observations in 150 goats were used to examine correspondence with individual feeding posture. The observed correspondence represents an association across recording stages rather than simultaneous validation of individual HFA/LFA phenotypes. Previous studies have reported high recognition performance for multiple behaviors in dairy goats and feeding-related behaviors in cattle using different YOLO architectures [14,31]. Compared with these studies, our model focused on the binary discrimination of HDF and NHDF but was evaluated under heterogeneous commercial-farm conditions characterized by variable illumination, image quality, and frequent occlusion by the feed barrier and neighboring animals. The lower *R* observed for HDF (79.74%) than NHDF (83.61%) likely reflects the greater difficulty of detecting the goat head when it was partially obscured during feeding. These findings emphasize that model performance should be interpreted in the context of both task complexity and environmental conditions rather than compared solely on numerical accuracy. Moreover, differences in illumination, background, camera position, and hardware across farms may limit cross-farm generalizability [32]. HDF, however, primarily captures head posture and an observable feeding state and should not be considered a direct measure of feed intake. Therefore, behavioral agreement alone cannot establish the production relevance of the HFA/LFA phenotypes. This limitation motivated exploratory comparisons of feed intake and milk production to examine their associations with the feeding-dynamics-based classification.

### 4.3. Exploratory Associations with Production and Physiological Indicators

Production relevance was explored at two distinct levels: pen-average DMI was assessed in the source pens, whereas milk yield and composition were measured individually in the selected 24-goat subset. The associations with DMI and milk yield suggest potential production relevance for the behavioral measures [33], although the lack of significant differences in milk composition does not establish equivalence between the groups. Under the relatively consistent dietary, lactational, and management conditions of the present study, milk composition may be subject to stronger physiological regulation, whereas differences in feeding activity may be more readily reflected in total nutrient intake and milk yield [34,35]. Consistent with this interpretation, the higher and more sustained feeding activity observed in goats from HFA pens was accompanied by greater nutrient intake, supporting the value of feeding dynamics as a behavioral phenotype related to production performance.

Beyond production traits, HFA and LFA goats also differed in selected serum physiological indicators. Goats from HFA pens showed higher IL-2 but lower TNF-α concentrations than goats from LFA pens. This pattern indicates differences in selected circulating immune-related indicators between the two groups; however, it should not be interpreted as evidence of enhanced or impaired immune function. IL-2 is involved in immune-cell activation and immune-response regulation, whereas TNF-α participates in inflammatory and immune processes [36,37,38]. However, given the single-time-point serum measurements and the lack of direct assessment of immune-cell populations or inflammatory pathways, these differences are more appropriately interpreted as physiological features associated with higher feeding activity rather than evidence of enhanced immune function in goats from HFA pens. Goats from HFA pens also exhibited higher CAT and T-AOC concentrations without a significant increase in MDA, suggesting a potentially more favorable systemic antioxidant profile [39,40]. Given the higher feed intake and milk yield of goats from HFA pens, the increased antioxidant capacity may reflect physiological adaptation to greater metabolic activity, although this interpretation requires further validation. Remarkably, several neuroendocrine-related indicators, including 5-HT, GABA, NPY, LEP, and BDNF, were higher in goats from HFA pens. Among these factors, NPY is an established orexigenic neuropeptide that promotes feed intake in sheep [41,42], whereas LEP is an adipocyte-derived hormone that conveys information on peripheral energy stores to the brain and plays a central role in the regulation of energy homeostasis and food intake [43,44]. 5-HT, GABA, and BDNF are also involved in the regulation of feeding behavior and energy balance [45,46,47]. The observed differences in these circulating indicators suggest an association between HFA/LFA classification and several neuroendocrine-related serum measures. However, because these indicators were measured only once in peripheral serum, the present results cannot establish a distinct neuroendocrine state, temporal physiological dynamics, central neural activity, or a causal direction between feeding behavior and neuroendocrine regulation.

### 4.4. Limitations and Future Directions

This study has several limitations that should be acknowledged. First, data were collected at a single commercial farm, and the detection datasets contained only 1075 and 1561 original annotated images. Augmentation increased training-image numbers but did not introduce independent recording conditions. Annotation-image selection also excluded empty scenes and images unsuitable for reliable annotation, limiting evaluation under these conditions. Date–pen grouping separated recording-source combinations but did not necessarily provide evaluation on previously unseen pens or animals. No additional external dataset or formal illumination- and occlusion-stratified performance assessment was available. Second, group-level observations covered only the first 60 min after feed delivery over three consecutive days and may not represent daily or longer-term feeding patterns. Individual HDF observations were obtained during a subsequent recording phase rather than the same feeding events. The data originated from only five pens, with individual observations nested within them, limiting independent between-pen replication. Head-posture measurements were also affected by unobservable periods, and excluding these periods does not establish the animals’ behavior while obscured. Third, HFA/LFA reference labels were derived from the temporal characteristics used to construct the RF features. Classification performance therefore reflects reproduction of the operational grouping rather than independent biological validity. Potential influences of body weight, social rank, and changes in feed composition were not disentangled, preventing attribution of the observed patterns solely to intrinsic feeding motivation. Finally, individual milk-production and serum assessments involved a selected subset of 24 apparently healthy goats, with selection partly informed by HDF duration. These comparisons have limited biological replication and representativeness and remain exploratory. DMI was measured at the pen level and cannot establish the intake of individual sampled goats. Single-time-point serum measurements cannot characterize within-animal physiological dynamics or establish causal relationships. Exclusion of animals with apparent health abnormalities also means that disease-detection performance was not evaluated. Future studies should include additional pens and farms, longer and coordinated behavioral observations, independent behavioral reference measures, repeated physiological sampling, and animals experiencing physiological transition or health disorders.

## 5. Conclusions

This study applied computer vision to automatically detect goats in the feeding area and recognize head-down feeding behavior from videos recorded at a commercial dairy-goat farm. In the evaluated dataset, RF-DETR achieved *mAP*@0.5 and *mAP*@0.5:0.95 values of 90.38% and 53.30%, respectively, for feeding-area goat detection, while the YOLO11-based HDF/NHDF model achieved 88.32% and 52.11%, respectively. The resulting occupancy time series provided interpretable feeding-dynamic measures, with RTFGC, FTFGC, and FGCIR contributing most to HFA/LFA classification. These outputs offer a basis for non-contact monitoring of post-feeding activity and may complement routine feeding-management observations. The production and serum comparisons in 24 goats remain exploratory, and single-time-point blood sampling does not support causal interpretation. Broader deployment will require independent validation across farms and longer observation periods, with further evaluation of continuous individual tracking and health-monitoring applications.

## Figures and Tables

**Table 1:** Composition and nutrient levels of the basal diet on a dry matter (DM) basis.

Ingredient	Content (%)	Nutrient Levels	Content
Alfalfa hay	17	Digestible energy ^2^ (MJ/day) DM	12.53
Oat hay	17	Crude protein (%)	16.71
Whole-plant corn silage	15.5	Ether extracts (%)	2.4
Beet Pulp	5.9	Neutral detergent fiber (NDF, %)	33.06
Soybean meal (43%)	9.8	Acid detergent fiber (ADF, %)	22.19
Cottonseed meal (46%)	6.7	Ash (%)	8.11
Distillers dried grains with solubles (DDGS, 25%)	2	Calcium (%)	0.71
Corn	24.3	Phosphorus (%)	0.39
NaCl	0.4		
NaHCO_3_	0.3		
MgO	0.1		
Ca(HCO_3_)_2_	0.2		
Limestone meal	0.3		
Premix ^1^	0.5		
Total	100		

Note: ^1^ The premix was included at 0.5% of the diet and supplied 15,000 IU vitamin A, 2200 IU vitamin D_3_, 50 IU vitamin E, 55 mg Fe, 12.5 mg Cu, 47 mg Mn, 24 mg Zn, 0.5 mg Se, 0.5 mg I, and 0.1 mg Co per kg of diet. ^2^ All nutrient values are based on calculated estimates.

**Table 2:** Training hyperparameters of the object detection models.

Hyperparameter	YOLO11	RF-DETR	RT-DETR	YOLO11 (Head Recognition)
Optimizer	SGD	AdamW	AdamW	SGD
Initial learning rate	0.01	0.01	0.0001	0.001
Learning rate schedule	Cosine annealing	Step decay	Step decay	Cosine annealing
Epochs	300	200	300	300
Batch size	16	8	8	32
Input image size (pixel)	640 × 640	640 × 640	640 × 640	640 × 640
Momentum	0.937	—	—	0.937
Weight decay	0.0005	0.0001	0.0001	0.0005
Workers	8	2	8	8
Loss function	Cls + Bbox + DFL	Cls + L1 + GIoU	Cls + L1 + GIoU	Cls + Bbox + DFL

Note: Group-level detection models were initialized with COCO-pretrained weights. Mosaic and MixUp augmentation were applied with a probability of 0.5, and early stopping was used with a patience of 50 epochs. For the individual head-down feeding behavior model, the first 10 backbone layers were frozen for 10 epochs and subsequently unfrozen for end-to-end fine-tuning. A fixed random seed of 42 was used during model training.

**Table 3:** Classification performance evaluation of random forest (RF) model.

Class	Accuracy (*Acc*, %)	Precision (*P*, %)	*F*1-Score (%)
Total	93.79	94.32	93.77
LFA	88.34	99.15	93.43
HFA	99.24	89.48	94.11

Note: HFA, high feeding activity goats; LFA, low feeding activity goats.

## Data Availability

The raw video recordings generated in this study are not publicly available because they were collected at a commercial farm and are subject to farm data-access restrictions and animal-management considerations. Processed data supporting the findings of this study, together with the analysis code and trained model weights, are available from the corresponding authors upon reasonable request, subject to applicable data-use restrictions.

## Supplementary Materials

The following supporting information can be downloaded at: https://www.mdpi.com/article/10.3390/ani16213390/s1. Figure S1: Architecture of the three deep learning models used for dairy goat detection. (A) Architecture of the YOLO11 model, including its backbone, neck, detection head, and key modules. (B) Architecture of the RF-DETR model, including the DINOv2 backbone and transformer decoder. (C) Architecture of the RT-DETR model, including the HGNetv2 backbone, feature fusion network, and detection decoder.
